# *Parachlamydiaceae* as Rare Agents of Pneumonia

**DOI:** 10.3201/eid0906.020613

**Published:** 2003-06

**Authors:** Gilbert Greub, Pierre Berger, Laurent Papazian, Didier Raoult

**Affiliations:** * Université de la Méditerranée, Marseille, France; † Hôpital Sainte Marguerite, Marseille, France

**To the Editor**: Members of the *Parachlamydiaceae* family are emerging intracellular bacteria living in amoebae (*1*,*2*). Serologic studies have suggested that *Parachlamydia acanthamoeba* might be an agent of community-acquired pneumonia transmitted from a water source (*3*,*4*). In a single occasion, 16s rRNA of a member of the *Parachlamydiaceae* family was amplified and sequenced from a bronchoalveolar lavage sample (*5*). Thus, to specify the role played by the *Parachlamydiaceae* as agents of lower respiratory tract infection, we developed a real-time polymerase chain reaction (PCR) assay and applied it to 1,200 bronchoalveolar lavage samples, taken mainly from patients with pneumonia of unknown cause and received in our diagnostic microbiology laboratory between 1997 and 2002.

DNA extraction was performed by using the MagNA Pure LC instrument and the MagNA Pure LC DNA Isolation Kit III (Roche Molecular Biochemicals, Mannheim, Germany). Real-time PCR was performed by using TaqMan technology and targeting the gene encoding for a nonmitochondrial ATP/ADP translocase (GenBank accession no. AF490592). This energy parasite gene is present only in rickettsiae, chlamydiae, and plant plastids (*6*). The master mixture was prepared from the TaqMan Universal Master Mix kit (Applied Biosystems, Foster City, CA), according to the manufacturer’s instructions, and included 200 nM of each primer (Adp81F 5´- TAGTGATCTGCTACGGGATTT, Adp84R 5´-TTGGATTAGGATATTGCAATTT) and 200 nM of the fluorescent labeled probe (6-FAM-5´-AACCTTGTAGAAGTAACCTGGAAGAACCAGC-3´-TAMRA, where 6-FAM is 6-carboxyfluorescein and TAMRA is 6-carboxytetramethylrhodamine). Amplification was carried out on the ABI 7700 sequence detection system (TaqMan system, Applied Biosystems), by running 45 cycles, with annealing temperature of 52°C and polymerization temperature of 60°C. To prevent carryover, 200 µ M of uracil triphosphate was part of the master mixture, and uracil-N-glycosylase was used systematically. *Parachlamydia acanthamoeba* strain Hall coccus (kindly provided by T.J. Rowbotham) (*3*) and sterile water were used as positive and negative controls, respectively. In addition, PCR was tested on *Chlamydophila pneumoniae* and *Chlamydia psitacci* and four strains of *Rickettsia*. All but one (*Rickettsia montana*) was negative, as were 64 sterile water controls.

Of the 1,200 broncheolar lavage samples tested, 5 (0.42%) were positive. When PCR was repeated for those five samples, four were negative for *P. acanthamoeba* DNA, and only one was a true positive, confirmed by sequencing the product of the additional PCR. The sequence shared 100% DNA homology with *P. acanthamoeba* strain Hall coccus (GenBank accession no. AF490592). The patient, a 31-year-old man who was HIV-positive, had pneumonia, cough, and no fever. Chest x-ray examination showed an opacity in the right lung and a bilateral infiltrate. Leukocyte count was 5,000/mm^3^ with 80 CD4 cells/mm^3^; microbiologic investigations (in which the bronchoalveolar lavage was examined for cytomegalovirus, *Chlamydophila pneumoniae*, *Legionella pneumophila*, *Pneumocystis carinii*, mycobacteria, and *Toxoplasma gondii*) did not identify a causal agent*.*

We developed a highly sensitive PCR, which could amplify as few as 10 bacteria/mL. The assay results in a relatively high specificity (1,195/1,199; 99.67%) because it uses a target gene found only in rickettsiae*,* chlamydiae, and plant plastids, and uses a specific DNA probe. We considerably decreased the risk of horizontal and vertical contamination of the PCR reaction by using uracil and uracil-N-glycosylase and by keeping reaction cups closed since the first amplification cycle.

More importantly, our study showed that *Parachlamydia* DNA is rarely found in bronchoalveolar lavage samples (0.083%). This suggests that persons are infrequently exposed to *Parachlamydia* organisms and, consequently, members of the *Parachlamydiaceae* seldom cause pneumonia in humans. In the only positive sample, whether *Parachlamydia* originated from bacteria in the oropharynx, from water, or from a colonization of the lower respiratory tract was not known; whether they caused the patient’s pneumonia is also not known. That two strains of *Parachlamydia* found in amoebae were recovered from the nasopharynx of healthy volunteers (*7*) favors the first hypothesis. However, that the positive broncholaveolar lavage specimen was taken from an HIV-positive patient with community-acquired pneumonia suggests that *Parachlamydia* might occasionally play a pathogenic role in AIDS patients. Moreover, any amoebae-associated bacteria should be considered as a potential emerging pathogen because intra-amoebal growth may lead to the selection of virulence traits and to the adaptation to professional phagocytes, such as alveolar macrophages (*1*,*2*). Further studies are warranted to determine whether *Parachlamydiaceae* causes community-acquired pneumonia, particularly in HIV-infected persons.
